# Low Responsiveness of Macroencapsulated Human Islets Towards Glucose Challenge Despite Excellent Survival in Silicone-Based Oxygen-Delivering Devices

**DOI:** 10.3390/bioengineering13010056

**Published:** 2025-12-31

**Authors:** Daniel Brandhorst, Heide Brandhorst, Daniel A. Domingo-Lopez, Eoin O’Cearbhaill, Fergal B. Coulter, Rebecca Spiers, Stefano Deotti, Helena M. Kelly, Garry P. Duffy, Paul R. V. Johnson

**Affiliations:** 1 Islet Transplant Research Group, Nuffield Department of Surgical Sciences, University of Oxford, Oxford OX3 9DU, UK; 2 Oxford Consortium for Islet Transplantation, Oxford Centre for Diabetes, Endocrinology, and Metabolism (OCDEM), Churchill Hospital, University of Oxford, Oxford OX3 9DU, UK; 3 Anatomy and Regenerative Medicine Institute (REMEDI), College of Medicine, Nursing and Health Sciences, National University of Ireland, H91 TK33 Galway, Ireland; 4 School of Mechanical Engineering, Centre for Biomedical Engineering, University College of Dublin (UCD), D04 V1W8 Dublin, Ireland; 5 School of Pharmacy & Biomolecular Sciences, Royal College of Surgeons in Ireland (RCSI), D02 VN51 Dublin, Ireland

**Keywords:** cell culture, cell therapy, encapsulation devices, humans, hypoxia, inflammation, islets of Langerhans, islet transplantation, oxygen supply

## Abstract

Islet encapsulation has the potential to enable transplantation without requirement for life-long immunosuppression. The period between implantation and revascularisation is most harmful for encapsulated islets as they receive nutrients and oxygen exclusively via diffusion. This critical time gap must be bridged with a temporary oxygen supply to prevent inflammation and apoptosis. Hence, we compared the efficiency of individual components of an oxygen-delivering matrix (hyaluronic acid (HA); HA + perfluorodecalin nanoemulsion; HA + perfluorodecalin nanoemulsion + oxygen) to provide a substitute for the extracellular matrix and to facilitate human islet survival. The islets were loaded into silicone-based macroencapsulation devices with multi-scale porous membranes designed to optimise revascularisation. Four to five days of normoxic culture revealed that non-oxygen-charged nanoemulsion prevented islet disintegration but did not reduce necrosis or apoptosis. Oxygen supply decreased the generation of reactive oxygen species and chemokines, thereby increasing islet yield. Stimulated insulin secretion of encapsulated islets was marginal and severely delayed. Islets incubated in oxygen-precharged nanoemulsion were characterised by the highest stimulation index. These data suggest that islet survival in macroencapsulation devices can be optimised with a multi-functional matrix providing mechanical support and temporary oxygen supply to reduce the production of pro-inflammatory mediators. Suitable oxygen delivery systems with an extended life span must identified before in vivo experiments can be undertaken.

## 1. Introduction

Transplantation (TX) of isolated islets of Langerhans is a well-established successful treatment option for patients with type 1 diabetes mellitus particularly for those patients suffering from hypoglycaemia unawareness [1]. According to the most recent report of the Collaborative Islet Transplant Registry (CITR), excellent islet graft function has been achieved in recipients, as determined by C-peptide secretion, HbA1c levels and the absence of severe hypoglycaemic events, but at the risk of severe adverse events induced by the lifelong treatment with potent immunosuppressive agents [2]. Islet encapsulation is one potentially viable option to overcome graft rejection without exposing patients to the life-threatening side effects of immunosuppression.

The possibility to implant and retrieve islet grafts if required for safety reasons, makes macroencapsulation devices an even more attractive option for clinical application. Compared with spherical microcapsules the geometry of macroencapsulation devices is generally characterised by a lower surface area-to-volume ratio which decreases the diffusion rate as exclusive driving force for the uptake and release of nutrients, insulin and oxygen as long as revascularisation and integration of the device into the implantation site is not completed [3,4,5,6,7].

The major goal of islet TX, to restore normoglycaemia and prevent patients with type 1 diabetes from hypo- and hyperglycaemia, can only be achieved when the most relevant specifications of the device are carefully balanced [8]. Amongst other factors, the device has to accommodate and keep a sufficient number of islets alive until the blood supply has been fully re-established [9]. Whilst allowing diffusion of nutrients and oxygen into the device, these technologies have to form an effective barrier against certain components of the host’s immune system, such as immune cells and antibodies, to prevent rejection of transplanted islets [10]. In addition, the encapsulation materials have to prevent or at least minimise fibrosis around the device [11,12,13].

Although the first studies about the immunoprotection of isolated tissue were undertaken several decades ago, most of the challenges associated with tissue encapsulation have not been solved and are still relevant for present trials [12,14]. For that reason, this study was designed as an initial in vitro test for an innovative oxygen-delivering macroencapsulation device with a multi-scale porous silicone-based coating [15,16] and for the identification of existing basic problems with the kinetics of human islet insulin release [17,18].

## 2. Materials and Methods

### 2.1. Human Islet Isolation

This study was approved by our institutional review board South Central—Oxford Research Ethics Committee (NHS Health Research Authority) using the reference number 09/H0605/2. Written informed consent was obtained from donor relatives.

Pancreases were retrieved from 15 (3 female/12 male) human multi-organ donors with brain death with a mean age of 48.1 ± 1.6 years (±standard error) and a mean body mass index of 28.7 ± 1.5 kg/m^2^. All pancreases were preserved with University of Wisconsin solution (Bridge to Life, London, United Kingdom (UK)) and had a mean cold ischaemia time of 6.4 ± 0.3 h. Human islets were isolated and purified using standard isolation techniques as previously described [19].

### 2.2. Beta-Shells

These macrodevices, called beta-shells, were produced by the Centre for Biomedical Engineering at the University College Dublin as previously described [15,16]. Briefly, fabrication involved deposition of an ink comprising emulsion of sodium chloride-saturated water, solvent-diluted silicone and a suitable Span/Tween surfactant blend. This was sprayed on to a heated plate, creating a membrane of 70 µm thickness. The membrane was then reinforced by depositing thin (150 µm) silicone filaments in a loopwise fashion (4 mm diameter) over the entire surface. After the silicone was cured, two pieces of the membrane were bonded together using an outline of extruded silicone along with a thin silicone inlet tube to create a pouch. Before use, the salt was removed by ultrasonic washing in deionised water and sterilised in an autoclave. The resulting device featured porous silicone coating (6–8 µm), characterised by a minimum wall thickness and high elasticity. The dimensions of this device, originally designed for rats, were 10 × 20 × 1.2 mm [16].

**Figure 1:**
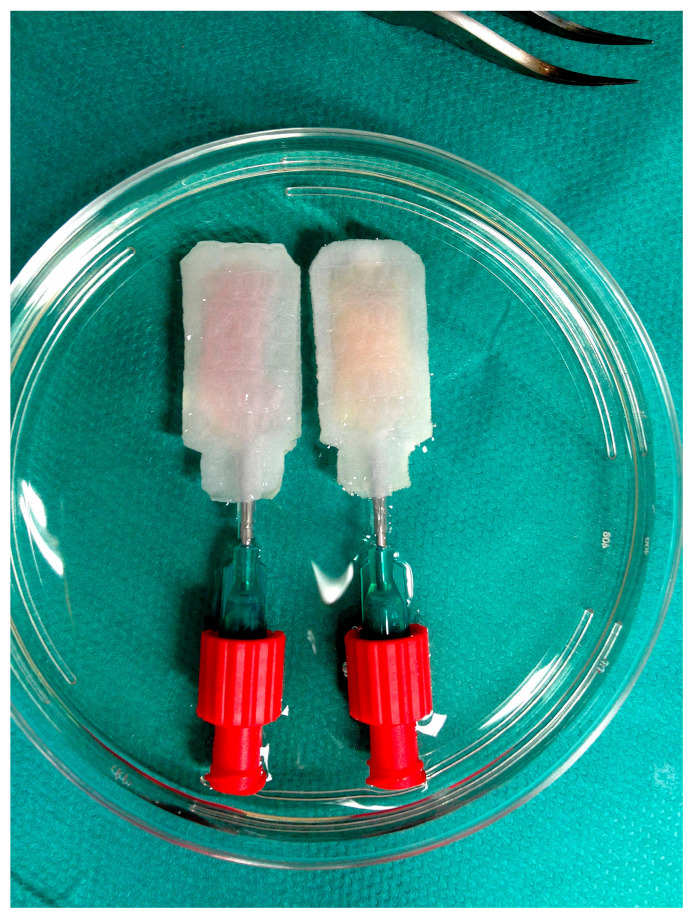
Two silicone-based multi-scale porous beta-shells manually infused with 600 human IEQ suspended in 300 µL of native HA-gel (left device) or O_2_-charged beta-gel (right device). The devices were immersed in 30 mL of supplemented CMRL 1066 and cultured for four to five days in normoxic atmosphere at 37 °C before islet characterisation.

An initial series of experiments (*n* = 5) was performed to test the charging capacity of the beta-shells by infusing either a low seeding density (S-Density), equivalent to 300 islet equivalents (IEQ), or a high S-Density, corresponding to 600 IEQ, which was dissolved in a volume of 300 µL of different media. Manual islet infusion was enabled by catheters attached to the inlets of the beta-shells (Figure 1).

As shown in Table 1, the experimental groups were designed in module-based manner using supplemented CMRL 1066 as control matrix and hyaluronic acid (HA-gel) as basis for the dispersion of the nano-emulsified oxygen carrier perfluorodecalin (PFD, beta-gel). The PFD emulsion and beta-gel formulation and oxygenation were carried out as previously described by Domingo-Lopez et al. [20]. Briefly, PFD nanoemulsions were prepared via ultrasonic processing using lipoid E80 as an emulsifier. These emulsions were incorporated into an HA-hydrogel by repeated shear mixing between two syringes to produce a homogeneous gel (beta-gel). The beta-gel was oxygenated by flushing oxygen (1.5 L min^−1^ O_2_) immediately before cell encapsulation or related applications to preserve oxygen content. Experimental groups involved in the initial beta-shell capacity study are indexed in Table 1.

### 2.3. Human Islet Culture

After charging, islet-loaded beta-shells were placed in 10 cm Petri dishes (Greiner Bio-One, Stonehouse, UK) filled with 30 mL of CMRL 1066 culture medium (PAN Biotech Ltd., Wimborne, UK) supplemented with 20 mmol/L HEPES, 2 mmol/L l-glutamine, 200 units/mL penicillin, 200 µg/mL streptomycin (all reagents from Life Technologies, Paisley, UK) and 10% foetal calf serum (PAA Laboratories, Pasching, Austria). In the first series of experiments, undertaken to assess the charging capacity of the beta-shells, 300 or 600 control IEQ were incubated free-floating in 8 mL of supplemented CMRL 1066 filled into 10 cm Petri dishes and compared with islets suspended in either 300 µL of supplemented CMRL or O_2_-charged beta-gel and infused into beta-shells. As shown in Table 1, the S-Density was 300 or 600 IEQ per 300 µL suspension volume, defined as low or high S-Density.

Free-floating and beta-shell-housed islets were cultured for four to five days in normoxic atmosphere (21% oxygen, 5% carbon dioxide) at 37 °C before postculture islet characterisation. After culture, the beta-shells were opened at the distal end by an incision and carefully flushed with 30 mL of supplemented CMRL 1066. The effluent was collected in conical 50 mL-tubes (Greiner Bio-One) and samples of islet-preconditioned medium were collected for subsequent measurement of cytokine release after centrifugation for 1 min at 100× *g*. After supernatant aspiration, the tissue was resuspended in 5 mL of fresh supplemented CMRL 1066 and processed for islet characterisation.

### 2.4. Islet Characterisation

Before and after culture, samples of defined volume were collected from any treatment group and processed to determine islet yield, apoptosis, morphological and membrane integrity, and production of pro-inflammatory mediators. Samples for islet counting were stained with dithizone (Sigma-Aldrich, Dorset, UK) to visually determine the number of actual islets (IN) defined as dithizone-positive size-independent insulin-containing cell clusters. Any counted islet was categorised according to its diameter and mathematically converted into IEQ considering the individual volume of counted islets as previously described [21]. This conversion was normalised to a “standard” islet of 150 µm in diameter, which was defined as one IEQ. The proportional IEQ yield (%) was normalised to the IEQ yield as counted preculture. Islet morphological integrity was determined by calculating the islet dissociation index dividing the number of size-independent IN by the IEQ (islet dissociation index = IN ÷ IEQ) [22].

Islet viability was assessed by utilising 0.67 µmol/L of fluorescein diacetate (FDA, Sigma-Aldrich) and 4.0 µmol/L of propidium iodide (PI, Sigma-Aldrich) for staining viable and dead cells, respectively [23]. IEQ overall survival was calculated considering the recovery of viable cells only, which were exclusively stained with FDA and not penetrated by PI. Early apoptosis was demonstrated by exclusive staining of phospatidylserine using 450 ng/mL annexin V FITC (Becton-Dickinson Biosciences, Oxford, UK). In contrast, islet late apoptosis was determined by simultaneous staining with annexin V and PI used at a concentration of 4.0 µmol/L, respectively [24,25]. Production of reactive oxygen species (ROS) was determined by measuring the intra-islet conversion of 60 µM dichlorofluorescein diacetate into fluorescent dichlorodihydrofluorescein as previously described [26]. The fluorescence intensity (FI) of FDA-PI, annexin V, and ROS was quantified utilising a fluorometric plate reader as previously described [27].

Pre- and postculture, islet-preconditioned cell-depleted supernatants were collected and assessed for secretion of hypoxia- and inflammation-related chemokines. Release of interleukin-1 beta (IL-1β), IL-6, IL-8, monocyte chemoattractant protein-1 (MCP-1), tumour necrosis factor alpha (TNF-α) and vascular endothelial growth factor A (VEGF-A) was measured by utilising enzyme immunoassays specific for human chemokines (all kits from Abcam, Cambridge, UK).

Islets intended for glucose stimulation were cultured in beta-shells for only 24 h at 37 °C before injection of 100 µL of 0.126 mol/L glucose solution into the devices. The final glucose concentration inside the beta-shells was 25 mmol/L. After glucose loading, the beta-shells were placed in 10 cm Petri dishes which were filled with 30 mL of supplemented CMRL with a glucose concentration of 5.5 mmol/L (Thermo Fisher, Basingstoke, UK). A duplicate baseline sample for insulin measurement was immediately taken from the incubation medium to define the start point of the incubation period for the following 150 min at 37 °C. Sampling was repeated after 75 and 150 min. The samples were stored at −20 °C before measurement utilising a human insulin-specific enzyme immunoassay from Mercodia (Uppsala, Sweden).

ROS production, islet chemokine release, glucose-stimulated insulin secretion, extent of necrosis and apoptosis were normalised to IEQ.

### 2.5. Statistical Analysis

Statistical analysis and graphical presentations were performed using Prism 10.5.0 (GraphPad, La Jolla, CA, USA). Data analysis was carried out using the nonparametric Friedman test followed by Dunn’s test for multiple comparisons or the Wilcoxon test for subsequent insulin release at 2 and 20 mmol/L of glucose. Parameters were related to IEQ and normalised to sham treatment or preculture data where appropriate. Differences were considered significant at *p*-values less than 0.05. *p*-values larger than 0.05 were termed nonsignificant (NS). The results are generally expressed as mean ± standard error (SEM).

## 3. Results

### 3.1. Assessment of Beta-Shell Storage Capacity

**Figure 2:**
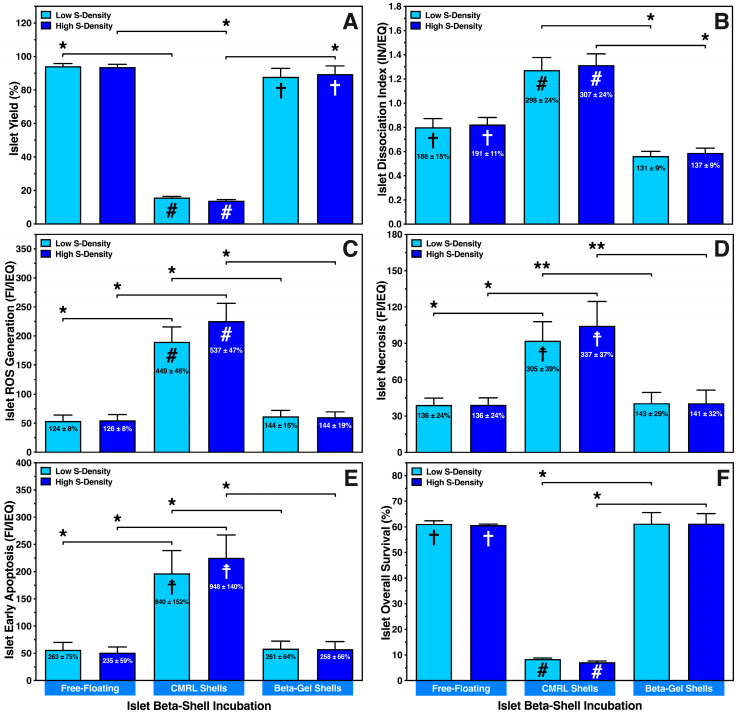
Effect of seeding density (S-Density) on characterisation of islets cultured at a density of 300 (low) or 600 (high) IEQ in silicone-based multi-scale porous macrodevices loaded with 300 µL of supplemented CMRL (CMRL shells) or O_2_-charged beta-gel (beta-gel shells). CMRL-incubated islets cultured in 8 mL of supplemented CMRL in 10 cm Petri dishes served as controls (free-floating). After 4–5 days of normoxic culture (*n* = 5) islets were characterised regarding (**A**) islet yield (%) normalised to islet preculture, (**B**) the islet dissociation index (IN/IEQ), (**C**) reactive oxygen species (ROS) generation (FI/IEQ), (**D**) islet necrosis (FI/IEQ), (**E**) islet early apoptosis (FI/IEQ), and (**F**) islet overall survival (%). Figures inside bars show data normalised to islet preculture. Symbols inside bars indicate # *p* < 0.001, ☨ *p* < 0.01, † *p* < 0.05 for comparison vs. islet preculture. Lines/arrows indicate * *p* < 0.05, ** *p* < 0.01 for comparison of experimental groups.

To evaluate the storage capacity of beta-shells, An amount of 300 IEQ, representing a low seeding density of 1 IEQ/µL, or 600 IEQ, equivalent to a high seeding density of 2 IEQ/µL, was suspended either in 300 µL of supplemented CMRL or O_2_-charged beta-gel when infused into the devices. These experimental groups were compared with free-floating islets serving as controls and cultured for 4–5 days at 37 °C in 10 cm Petri dishes. As shown in Figure 2A, free-floating islet culture yielded 94 ± 2% of the initially infused islet mass regardless of whether a low (300 IEQ) or high (600 IEQ) seeding density of islets had been incubated. In contrast, 4–5 days of culture in the devices resulted in a significant loss of islets (*p* < 0.05 vs. free-floating) of more than 80% (low: 16 ± 1%; high: 14 ± 1%) when supplemented CMRL was used for islet matrix. This massive reduction could be ameliorated by replacing CMRL with O_2_-charged beta-gel and then reaching a similar level of recovery as free-floating islets. Again, no significant difference was found between low (88 ± 5%) and high (90 ± 5%) islet load.

The same applies to all exp. groups when the islet dissociation index (preculture: 0.43 ± 0.01 IN/IEQ) was measured (Figure 2B). After culture under free-floating conditions or in CMRL-filled beta-shells, islet dissociation was nearly doubled (*p* < 0.05 vs. preculture) or tripled (*p* < 0.001 vs. preculture; *p* < 0.05 vs. O_2_-charged beta-gel), respectively. In contrast, when beta-shells were loaded with O_2_-charged beta-gel, the postculture dissociation index was in a similar range as in islet preculture (low: 131 ± 9%, NS; high: 137 ± 9%, NS). The significant dissociation and loss of human islets in CMRL-filled beta-shells corresponded to a four- to five-fold enhancement of the initial reactive oxygen species (ROS) production (preculture: 43.4 ± 6.6 FI/IEQ) as shown in Figure 2C (low: 449 ± 46%, high: 537 ± 47%, *p* < 0.001 vs. preculture). A substantial increase in ROS was also found when islets in CMRL-filled beta-shells were compared with islets cultured under free-floating conditions or beta-shells loaded with O_2_-charged beta-gel (*p* < 0.05). A marginally higher ROS generation (NS) could be observed when a higher seeding density was used in CMRL-filled beta-shells, whilst the ROS production in free-floating islets (124 ± 8% vs. 126 ± 8%, NS) and islets encapsulated in beta-gel shells (144 ± 16% vs. 144 ± 19%) were nearly identical (Figure 2C).

As demonstrated in Figure 2D, the increased ROS production also seemed to correlate with islet necrosis (preculture: 34.3 ± 8.6 FI/IEQ) in CMRL-filled beta-shells (*p* < 0.01 vs. preculture). In contrast, islets encapsulated in O_2_-charged beta-gel showed significantly reduced necrosis (*p* < 0.01 vs. CMRL-filled beta-shells), reaching a similar level as found in islet preculture. Agreeing with these findings, a very tight correlation with early apoptosis was noted following the same pattern as ROS (Figure 2E). However, whilst early apoptosis was expressed preculture at a low level (24.1 ± 2.5 FI/IEQ), massive increases of 840 ± 152% and 948 ± 140% (*p* < 0.01 vs. preculture; *p* < 0.05 vs. free-floating, O_2_-charged beta-gel shells) after low and high islet load into CMRL-filled beta-shells, respectively. No difference in early apoptosis was found between free-floating islets and islets in O_2_-charged beta-gel shells (Figure 2E).

IEQ overall survival, considering the recovery of viable cells only, followed the same trend that could be observed for all other parameters assessed: islets cultured under free-floating conditions survived at a very similar level as islets cultured in O_2_-charged beta-gel shells, whilst islets cultured in CMRL-filled beta-shells had an overall survival below ten percent (*p* < 0.05 vs. O_2_-charged beta-gel shells), indicating an unfavourable and non-physiological microenvironment (Figure 2F).

### 3.2. Maintenance of Islet Integrity in Beta-Shells

The second trial of this study aimed to identify the matrix component that is most impactful for islet survival in a microenvironment characterised by limited space and oxygen supply. For that purpose, the efficiencies of HA-gel, HA-gel plus PFD emulsion (beta-gel) and HA-gel plus PFD emulsion plus oxygen (beta-gel + O_2_) to protect islet integrity were compared with supplemented CMRL serving as control medium (Table 1).

**Figure 3:**
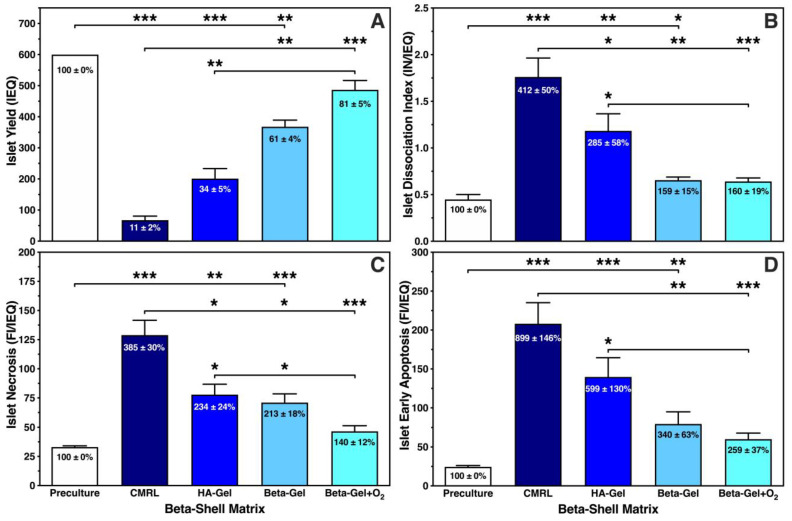
Effect of different matrix components on 600 IEQ cultured for 4–5 days in silicone-based multi-scale porous macrodevices incubated under normoxia (*n* = 10). Devices were loaded with 300 µL of supplemented CMRL, hyaluronic acid (HA)-gel, beta-gel, or O_2_-charged beta-gel. Islet characterisation included (**A**) islet yield (IEQ), (**B**) islet dissociation index (IN/IEQ), (**C**) islet necrosis (FI/IEQ), and (**D**) islet early apoptosis (FI/IEQ). Figures inside bars show data normalised to islet preculture. Lines/arrows indicate * *p* < 0.05, ** *p* < 0.01, *** *p* < 0.001 for comparison of experimental groups.

As demonstrated in Figure 3A, a substantial reduction in islet yield was noted in any experimental group except when islets had been cultured in O_2_-charged beta-gel shells. The islet yield dropped by nearly 90%, 70% or 40% when islets were immersed in supplemented CMRL, HA-gel (*p* < 0.001 vs. preculture) or non-oxygenated beta-gel (*p* < 0.01 vs. preculture), respectively. Despite an islet recovery of 81 ± 5%, no significant decrease in yield was noted postculture after encapsulation in O_2_-charged beta-gel shells. Islet loss was partially associated with islet morphological dissociation during 4–5 days of culture. As shown in Figure 3B, the postculture dissociation index was lowest in beta-shells loaded with beta-gel either O_2_-charged (0.64 ± 0.04; NS vs. preculture) or not (0.65 ± 0.03; *p* < 0.05 vs. preculture). Again, most abundant islet dissociation was observed when islets had been cultured in CMRL-filled beta-shells (*p* < 0.05 vs. HA-gel; *p* < 0.01 vs. non-charged beta-gel; *p* < 0.001 vs. preculture and O_2_-charged beta-gel). Islet mortality, determined as either necrosis (Figure 3C) or early apoptosis (Figure 3D), was extensively present in islets infused into CMRL-filled beta-shells. A massive increase in islet necrosis and early apoptosis was noted compared with islets preculture, reaching a level of 385 ± 30% (*p* < 0.001 vs. preculture, and non-charged beta-gel, O_2_-charged beta-gel) and 899 ± 146% (*p* < 0.001 vs. preculture and O_2_-charged beta-gel; *p* < 0.01 vs. non-charged beta-gel) in CMRL-filled beta-shells, respectively.

**Figure 4:**
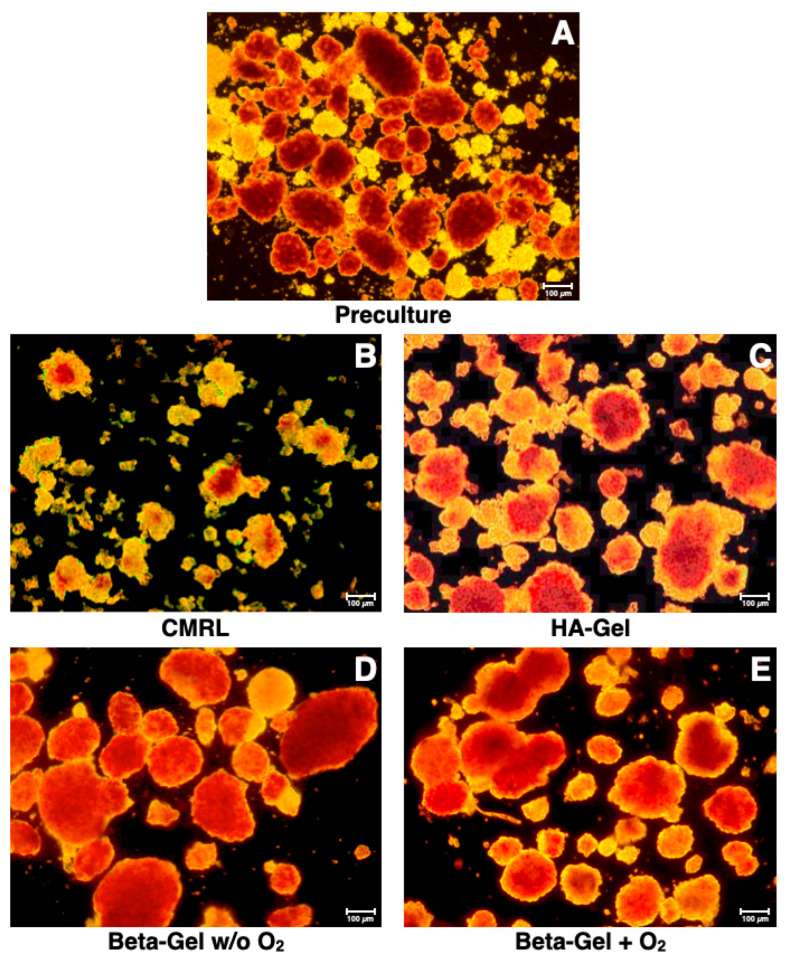
Effect of different matrix components on the morphological integrity of dithizone-stained islets cultured for 4–5 days in silicone-based multi-scale porous macrodevices incubated under normoxia. All treatment groups were isolated from the same donor pancreas. Images are representative of the entire series of experiments (original magnification ×50). Islets isolated before starting the culture experiments are shown in (**A**). Devices were loaded with 300 µL of (**B**) supplemented CMRL, (**C**) hyaluronic acid (HA)-gel, (**D**) beta-gel, or (**E**) O_2_-charged beta-gel initially containing 600 IEQ.

The morphology of islets cultured in different beta-shell-housed matrices is demonstrated in Figure 4. The structure of islets incubated in supplemented CMRL (Figure 4B) was defined by an irregularly shaped periphery and a weak staining with dithizone. In addition, numerous islet fragments were harvested from supplemented CMRL reflecting the massive loss of islets after 4–5 days of culture (Figure 3A). When HA-gel served as the islet matrix in beta-shells, the islet size was increased compared with supplemented CMRL, but islet morphology was still characterised by an irregular periphery (Figure 4C). In contrast, islets cultured in non-charged (Figure 4D) or O_2_-charged beta-gel (Figure 4E) were characterised by an average similar size, as reflected by the dissociation index (Figure 3B). Both treatments resulted in a strong dithizone stain as well as an ovoid and regular shape (Figure 4D,E) but only islets incubated in O_2_-charged beta-gel had a morphological integrity that was not significantly different compared with islet preculture (Figure 3B and Figure 4A).

### 3.3. Anti-Inflammatory Efficiency of Different Beta-Shell Matrices

**Figure 5:**
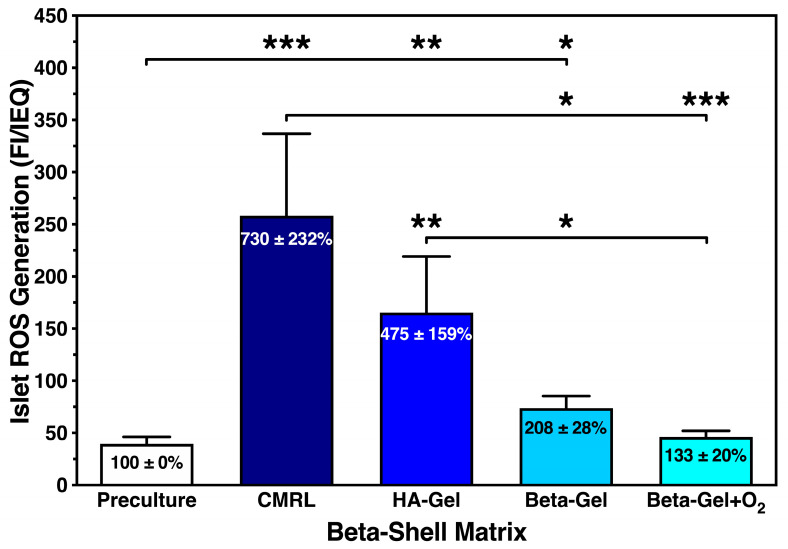
Effect of different matrix components on the reactive oxygen species (ROS) generation of islets cultured for 4–5 days in silicone-based multi-scale porous macrodevices incubated under normoxia (*n* = 10). Devices were loaded with 300 µL of supplemented CMRL, hyaluronic acid (HA)-gel, beta-gel, or O_2_-charged beta-gel. Figures inside bars show data normalised to islet preculture. Lines/arrows indicate * *p* < 0.05, ** *p* < 0.01, *** *p* < 0.001 for comparison of experimental groups.

Compared with islet preculture, storage in beta-shells induced a massive increase in the ROS production in cultured islets (Figure 5). This mainly concerned islets suspended in CMRL (730 ± 232%; *p* < 0.001 vs. preculture and O_2_-charged beta-gel; *p* < 0.05 vs. non-charged beta-gel) and HA-gel (475 ± 159%, *p* < 0.01 vs. preculture, O_2_-charged beta-gel; *p* < 0.05 vs. non-charged beta-gel). In contrast, islets cultured in O_2_-charged beta-gel showed only a marginal increase in ROS generation (133 ± 20%; NS vs. preculture) when compared with islet preculture. As displayed in Figure 5, the waiving of O_2_-charging in beta-gel resulted in almost doubling of the ROS production compared with islet preculture (208 ± 28%; *p* < 0.05 vs. preculture). Since ROS is the key mediator of TNF-α-induced activities, we also assessed the release of other members of the TNF-α-dominated chemokine network.

**Figure 6:**
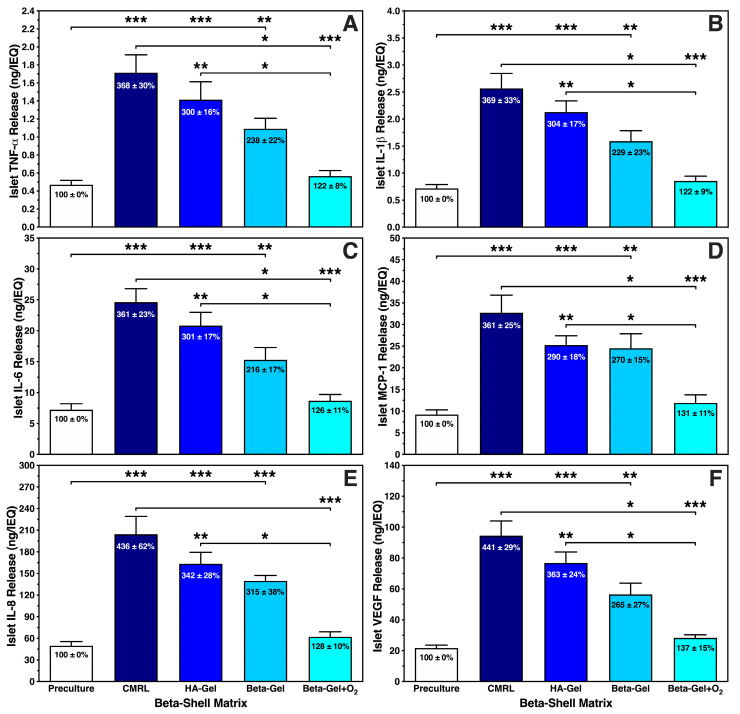
Effects of different matrix components on the production of (**A**) TNF-α, (**B**) IL-1β, (**C**) IL-6, (**D**) MCP-1, (**E**) IL-8, and (**F**) VEGF-A released by islets during 4–5 days of normoxic culture in silicone-based multi-scale porous macrodevices (*n* = 10). Devices were loaded with 300 µL of supplemented CMRL, hyaluronic acid (HA)-Gel, beta-gel, or O_2_-charged beta-gel initially containing 600 IEQ. Figures inside bars show data normalised to islet preculture. Lines/arrows indicate * *p* < 0.05, ** *p* < 0.01, *** *p* < 0.001 for comparison of experimental groups.

Figure 6 demonstrates that the different chemokines were secreted in a very similar pattern. The incremental reduction in each chemokine correlated with the complexity of the medium used as matrix. Islets cultured in CMRL-filled beta-shells showed a nearly four-fold increase of approximately 370% (*p* < 0.001 vs. preculture and O_2_-charged beta-gel) when measuring TNF-α (Figure 6A), IL-1β (Figure 6B), IL-6 (Figure 6C), and MCP-1 (Figure 6D). A slightly higher release of approximately 440% (*p* < 0.001 vs. preculture and O_2_-charged beta-gel) was detected for IL-8 (Figure 6E) and VEGF-A (Figure 6F). Islets suspended in HA-gel released the triplicate amount of TNF-α, IL-1β, IL-6 and MCP-1 (Figure 6A–D) when compared with islet preculture (*p* < 0.001 vs. preculture; *p* < 0.01 vs. O_2_-charged beta-gel) whilst the incremental increase in IL-8 and VEGF-A was approximately 350% (Figure 6E,F) compared with islet preculture (*p* < 0.001 vs. preculture; *p* < 0.01 vs. O_2_-charged beta-gel). As similarly observed with ROS, islets immersed in O_2_-charged beta-gel secreted the lowest quantity of chemokines varying between 122 ± 8% for TNF-α and 137 ± 15% for VEGF-A when compared with islet preculture (NS). When beta-gel was not charged with oxygen, this amount was nearly doubled reaching approximately 240% increase when normalised to islet preculture. The highest incremental increases were found in IL-8 (315 ± 38%) and VEGF-A (265 ± 27%).

**Figure 7:**
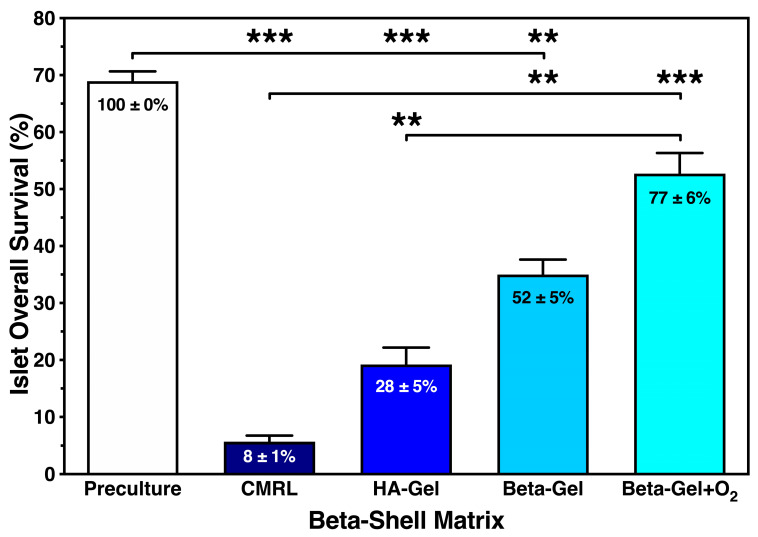
Effect of different matrix components on the overall survival of islets cultured for 4–5 days in silicone-based multi-scale porous macrodevices incubated under normoxia (*n* = 10). Devices were loaded with 300 µL of supplemented CMRL, hyaluronic acid (HA)-gel, beta-gel, or O_2_-charged beta-gel initially containing 600 IEQ. Figures inside bars show data normalised to islet preculture. Lines/arrows indicate ** *p* < 0.01 and *** *p* < 0.001 for comparison of experimental groups.

We calculated the islet overall survival equivalent to the recovery of only viable cells to combine the anti-inflammatory potencies of the different beta-shell matrices. As presented in Figure 7, islet overall survival was marginal when CMRL was used for islet culture/storage in beta-shells, reaching a survival rate of less than 10% compared with islet preculture (*p* < 0.001 vs. preculture and O_2_-charged beta-gel; *p* < 0.01 vs. non-charged beta-gel). A five-fold higher survival rate was observed in non-charged beta-gel (*p* < 0.01 vs. preculture and CMRL). Approximately one quarter of the initially infused islet yield was lost during culture when islets were immersed in O_2_-charged beta-gel (NS vs. preculture; *p* < 0.001 vs. CMRL; *p* < 0.01 vs. HA-gel).

**Figure 8:**
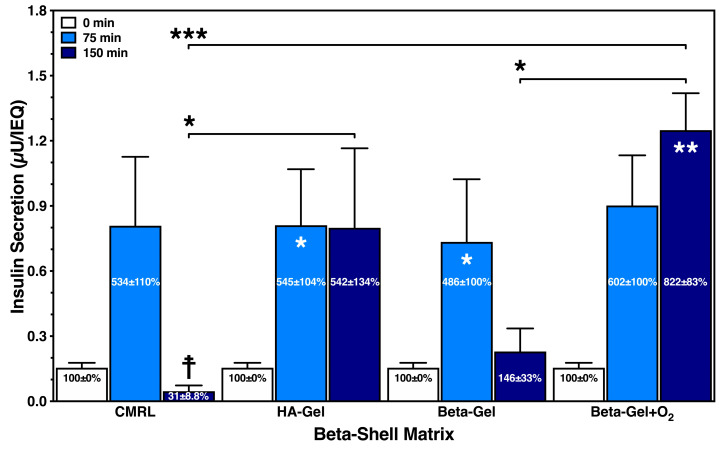
Effect of different matrix components on the glucose-stimulated insulin secretion of islets cultured for 24 h in silicone-based multi-scale porous macrodevices incubated under normoxia (*n* = 4). Devices were loaded with 300 µL of supplemented CMRL, hyaluronic acid (HA)-gel, beta-gel, or O_2_-charged beta-gel initially containing 600 IEQ. Figures inside dark blue bars show insulin release when normalised to time point zero. Indices indicate * *p* < 0.05, ** *p* < 0.01 vs. 0 min, and ☨ vs. 75 min. Arrows indicate * *p* < 0.05 and *** *p* < 0.001 for comparison of experimental groups at 150 min.

As demonstrated in Figure 8, the administration of 25 mmol/L of glucose resulted in an extremely low release of insulin per IEQ. The glucose stimulation index accumulated to approximately 500% after 75 min in all treatment groups, except in islets cultured in O_2_-charged beta-gel shells, producing an approximately six-fold increase in the initial baseline insulin content in the medium. After 150 min of incubation, a substantial drop in stimulated insulin release was measured in islets cultured in supplemented CMRL and non-oxygenated beta-gel. Whilst islets cultured in HA-gel shells secreted a very similar amount of insulin after 75 and 150 min, islets incubated in O_2_-charged beta-gel shells showed a strong accumulative effect on insulin release after 150 min.

## 4. Discussion

The enormous challenges that have been associated with the immunoprotection of isolated islets, as already shown by initial studies in this field [12,14], remain significant in current studies. The design and construction of an efficient macrodevice must maintain a delicate balance between efficient immunoprotection and an adequate entrance of nutrients and oxygen to keep islet cells alive until revascularisation of the device is established. The size of the device determines its capacity to accommodate a sufficient islet load to restore normoglycaemia in the patient [6,28]. Meanwhile, the volume of the device should not be in conflict with a physiological insulin response towards increasing glucose levels [3,4]. To cover these multiple and partially conflicting requirements, the design of macrodevices should allow for the monitoring of graft viability and survival followed by feedback with respect to replenishment of nutrients, oxygen, and cells [29]. This study was designed as an initial in vitro test to collect relevant data about a macrodevice’s efficiency and identify its weak points to technically optimise construction before assessment in vivo.

### 4.1. Beta-Shell Storage Capacity

The initial storage capacity experiments in our study revealed that beta-shells filled with O_2_-charged beta-gel matrix can take a load of 2 IEQ per µL without any disadvantages when compared with free-floating islets (0.075 IEQ/µL) or with a lower seeding density of 1 IEQ/µL. The latter seeding density corresponds with our clinical protocol, where a maximum of 25,000 IEQ is immersed in a volume of 25 mL of medium per culture flask. This seeding density provides nearly ideal conditions when islets are seeded into 175 cm^2^ T-flasks stored in an incubator before transplantation into a patient [30]. Nevertheless, when beta-shells were filled with supplemented CMRL, the overall survival of cultured islets dropped by more than 90%. The high expression of necrosis and apoptosis in islets loaded into CMRL shells correlated with the high production rate of ROS. The doubling of the seeding density from 1 to 2 IEQ/µL had a stimulatory effect on the islet ROS production in CMRL shells, which was not the case in O_2_-charged beta-gel.

Our initial findings were confirmed in the main part of this study assessing the effect of different matrix components on islet survival. It became evident that a highly viscous matrix, such as the beta-gel, provided a solid mechanical support that was beneficial for islet recovery even when it had not been charged with oxygen. It can be assumed that a solid matrix is more effective in preventing disintegration of embedded islets than liquid matrices such as CMRL and hyaluronic acid [31]. Meanwhile, an oxygen carrier such as perfluorodecalin (PFD) is characterised by a high affinity toward oxygen and may easily absorb this gas from the local environment, thereby promoting cell death in encapsulated islets. Consequently, ROS and chemokines were significantly elevated in non-charged beta-gel when compared with O_2_-charged beta-gel, which had implications for the other determinants of islet integrity and mortality such as yield, necrosis, and apoptosis. This is in broad agreement with previous findings demonstrating that an inadequate supply of oxygen to isolated islets results in the activation of genes [32,33,34] associated with the activation of pro-inflammatory and pro-apoptotic pathways [27,35,36,37].

### 4.2. Islet Oxygen Supply

Depending on the islet size, several mathematical models have been used to calculate the maximum seeding density under the consideration that the islet core must be supplied with oxygen to preserve the morphological and functional integrity of an islet preparation sufficient to cure a patient with type diabetes [20]. However, potential transplant sites that are attractive for implanting and retrieving macrodevices for safety reasons, such as the intramuscular or subcutaneous site, have local oxygen pressures that are too low to penetrate the device membranes and reach the islet core via passive diffusion [6]. The large dimensions of a macrodevice that could provide these conditions are physically incompatible with being implanted into a patient [5,9,38]. Moreover, these models remain too optimistic because they integrate only pure islet preparations into the calculations, which is unrealistic. Using isolated pig islet preparations, it has been shown that digested acinar tissue, contaminating the islets after purification, is characterised by an ongoing oxygen consumption, which is temporarily higher than in isolated islets [39].

The implementation of oxygenation techniques within the device, or in close proximity to it, appears to be the most reasonable strategy to bridge the time gap between device implantation and completed revascularisation [6,29]. The Beta-O_2_ device is an example of a device that can successfully preserve the integrity of densely packed allo-transplanted islets in a patient with type 1 diabetes over months with continuous oxygen supply from an attached oxygen chamber with a limited capacity which requires daily refilling [40,41]. Other approaches that incorporate oxygen carriers in the devices have suffered from the same limitation [42,43,44]. Similarly, our approach used a PFD nanoemulsion as an oxygen carrier that absorbs oxygen up to 475–641 torr; however, its working capacity lasts for approximately 90 h [20]. This time span can cover the initial phase of islet revascularisation but cannot bridge the time until full revascularisation and engraftment of transplanted islets is obtained [45,46,47]. This applies to islets encapsulated in a macrodevice where newly formed vessels must connect to the outer membranes. The multi-scale porous engineering of our macrodevice membranes significantly increased the density of membrane-attached vessels several-fold and promoted integration of the device into the graft site of rats, as shown in a previous study [15]. A further boost to the revascularisation and vascularity of the device was obtained by the local release of VEGF [16]. Most recent studies identified bFGF as an efficient promoter of islet revascularisation in the subcutaneous graft site [48].

Nevertheless, the successful prevascularisation of an empty device cannot promote the internal revascularisation of encapsulated islets, which remains characterised by a lack of oxygen and the mandatory need to implement an oxygen-delivering source [6]. A more optimistic outlook can be given for future trials, since innovative and convenient techniques are now available that can chemically produce oxygen over a prolonged period of time, covering at least the period until internal islet revascularisation is completed [6,44,49]. By balancing several essential variables such as local atmosphere, seeding density, islet viability, oxygen carrier, and oxygen generator via statistical modelling, it was shown that the careful combination of a potent oxygen donor and a suitable oxygen carrier with anti-oxidant characteristics, which prevents side effects of hyperoxia, can increase the seeding density nearly ten-fold to 3300 IEQ/cm^2^ without affecting islet viability, thereby reducing the required dimensions of a macroencapsulation device [28].

### 4.3. Anti-Inflammatory Potency

Hypoxia is directly linked to inflammation [50]. The central pivot for hypoxia-induced pro-inflammatory pathways is the mitochondrion [51]. Hypoxia activates HIF-1α, which reverses electron transfer in the mitochondria towards glycolysis, which in turn increases ROS as a byproduct. Under physiological conditions, small amounts of ROS are buffered by anti-oxidants and anti-oxidant enzymes [52,53]. In the case of continuous overproduction, ROS cannot be neutralised anymore due to the islet-specific low expression of anti-oxidant enzymes such as superoxide dismutase, catalase, and glutathione peroxidase [54,55,56]. The excessive release of ROS during prolonged hypoxia results in the NFκB-mediated activation of the chemokine network, which is dominated by TNF-α. Previous studies have clearly demonstrated that NFκB also upregulates several pro-apoptotic genes [32,53,57]. Consequently, a self-amplification pathway is initiated where TNF-α and other chemokines enhance the production of ROS, which is associated with the release of cytochrome-C as the starting point for the caspase cascade [58,59,60].

Our findings demonstrate that islets embedded in the O_2_-charged matrix in beta-shells produced significantly less ROS and chemokines compared with all other experimental groups. Our data revealed that not only pro-inflammatory and pro-apoptotic but also pro-angiogenic and protective chemokines, such as IL-8 [61] and VEGF-A [62], were decreased to a similar level to that of preculture status. Consequently, the preventive effect of oxygen significantly reduced islet mortality as expressed by the lower rates of necrosis and early apoptosis. Nonetheless, the working capacity of our oxygen carriers lasted for approximately only 90 h and required an advancement to an extended oxygen-supplying capacity towards a period of at least 14 days, which more or less covers the phase of complete revascularisation of islets, as discussed in Section 4.2. This could be achieved by including reoxygenation strategies applied via external refuelling or using oxygen-producing materials. Nevertheless, this strategy would essentially require separating the oxygen-carrying material from the HA-based matrix.

The anti-inflammatory potency may also have relevant implications for the foreign body response (FBR), which is an inflammatory and wound-healing response of the recipient’s body towards the implantation of a device [63]. After vascular damage during surgical implantation, the FBR is initiated by the binding of plasma proteins, fibrinogen and complement and is finalised by the formation of a collagen- and laminin-integrating capsule that encloses the implanted device. Macrophages are the most relevant cell population involved in the beginning and finalisation of the FBR [64]. At the onset of this process, macrophages are recruited by chemokines secreted from neutrophils [12]. Nevertheless, hypoxic islets express a pro-inflammatory gene profile that perpetuates the release of ROS and chemokines, which substantially extend the attraction of macrophages and other pro-inflammatory cell populations [27,34,65,66]. Hence, the release of islets from hypoxia via oxygenation is an effective strategy to increase islet survival and minimise the FBR.

### 4.4. In Vitro Function of Macroencapsulated Islets

This study demonstrated that the glucose-stimulated secretion of insulin is extremely low and severely delayed when islets are placed into macrodevices such as beta-shells. Compared with non-encapsulated human islets, the stimulated insulin release was reduced in a range between 95 and 90% [67]. This observation might be explained by the geometry of macrodevices, which are generally characterised by a lower surface area-to-volume ratio compared with spherical microcapsules. This low ratio decreases the diffusion rate of nutrients, insulin, and oxygen [3,5], which are driven by concentration gradients that have to build up via local accumulation until a steep gradient is formed for glucose entry and afterwards for insulin to leave the device [4]. Although we injected a large glucose bolus directly into the devices to immediately establish a strong gradient for glucose, the release of insulin from the device was severely delayed in relation to the timing of stimulation. In this setting, the amount of insulin released after 75 min was similar in all experimental groups. Differences between treatments became obvious after 150 min of incubation when a significant drop in insulin release was found in beta-shells loaded with supplemented CMRL or non-oxygenated beta-gel, whilst an ongoing continuous insulin release was measured in islets incubated in HA-gel. Moreover, when beta-gel shells were O_2_-charged, the amount of insulin released after 150 min was the highest amongst all other experimental groups, exceeding that of the previous time point by approximately one-third and that of non-oxygenated beta-gel by nearly six-fold. The latter observation may be partially explained by non-oxygenated PFD’s strong ability to uptake oxygen remaining in the local environment of the islets [68,69]. It can be assumed that the absorption of oxygen may transform a hypoxic environment into an anoxic one, thus blocking any secretory activity of islets [70].

As discussed in the upper sections, the presence of sufficient oxygen is vital to prevent the activation of NFκB and HIF-1α, which impairs glucose uptake and glucose sensing, thereby disturbing the physiological glucose-stimulated insulin responsiveness [32,33,71]. However, even if an effective oxygen source is present to diminish hypoxic stress and preserve islet graft integrity, this does not guarantee a physiological graft function to level postprandial blood glucose to normoglycaemic ranges, as shown in a patient with type 1 diabetes [41,72]. In contrast, when the Beta-O_2_ device was transplanted into streptozotocin-injected diabetic rats, normoglycaemia was measured throughout the entire observation period [40]. This finding was confirmed by another study with the same animal model demonstrating normoglycaemia in streptozotocin-injected diabetic rats after transplantation of macro-encapsulated islets despite the absence of a glucose-stimulated insulin response from the graft [73]. This discrepancy between human recipients and rodents can be partially explained by experiments with diabetic rats providing strong evidence that normoglycaemia can be achieved after glucose challenge of rats without involvement of insulin. Glucose’s ability to stimulate its own uptake and suppress its endogenous production has been termed glucose effectiveness but has only been discussed once in the context of encapsulated islet transplantation in rodents [74].

As long as a device is not connected to the vascular system of the graft site, insulin release mainly depends on a strong gradient that promotes the diffusion of insulin outside the device. For this reason, the local accumulation of stimulated insulin inside the device is essential to form an adequate diffusion gradient for insulin exit [4]. Nevertheless, if the insulin accumulation reaches a critical threshold inside the device, then a negative feedback signal is initiated in the islets completely blocking further insulin secretion [75]. Although the negative insulin feedback loop was described for islet tissue decades ago [76,77,78], this mechanism has only sporadically been disputed in the context of stimulated insulin release from macro-encapsulated islets [3].

Regarding the geometric design of macrodevices there is little space for optimisation of the delicate balance between islet load, insulin accumulation, gradient-driven insulin diffusion, and negative insulin feedback [38]. Prevascularisation may be the most efficient way to solve this dilemma implemented in the concept of extravascular macroencapsulation devices [79]. When our device was implanted and integrated into the submuscular site of diabetic pigs and filled with insulin infusion solution, the decrease in blood glucose was nearly identical to that with subcutaneous insulin injections into the recipients [15]. Nevertheless, as discussed in the Discussion Section 4.2, the time gap for internal revascularisation is characterised by severe hypoxia which requires a temporary and supplementary generation of oxygen until islet revascularisation is completed [6].

## 5. Conclusions

This study investigated an innovative oxygen-delivering macroencapsulation device that is coated with silicone-based multi-scale porous membranes that promote revascularisation and integration into the graft site. Our data suggest that islet survival inside the device can be significantly optimised with a multi-functional matrix that provides (I) a substitute for the digested extracellular matrix, (II) mechanical support to prevent islet disintegration, and (III) temporary oxygen supply to reduce the production of pro-inflammatory mediators such as ROS and chemokines resulting in decreased islet necrosis and apoptosis. Despite its anti-inflammatory efficiency to increase islet survival, the matrix did not prevent marginal and severely delayed glucose-stimulated insulin release from the device, which requires the integration of oxygen donors with extended life span to bridge the time gap between implantation and completed revascularisation.

## Figures and Tables

**Table 1:** Experimental groups.

Exp. Group	Device	Matrix	PFD Emulsion	Oxygen	Loaded Vol. (mL)	Infused Islets (IEQ)
Free-Floating ^a^	Petri Dish	CMRL	–	–	8	300/600
CMRL ^a^	Beta-Shell	CMRL	–	–	0.3	300/600
HA-Gel	Beta-Shell	HA-Gel	–	–	0.3	600
Beta-Gel	Beta-Shell	HA-Gel	+	+	0.3	600
Beta-Gel + O_2_^a^	Beta-Shell	HA-Gel	+	+	0.3	300/600

CMRL, supplemented CMRL 1066; HA, hyaluronic acid; PFD, perfluorodecalin; Vol., volume. ^a^ Included in the Beta-Shell Storage Capacity study.

## Data Availability

The raw data supporting the conclusions of this article will be made available by the authors on request.
